# Interactive effect of socio-eco-demographic characteristics and perceived physical activity barriers on physical activity level among older adults

**DOI:** 10.1186/s11556-022-00288-y

**Published:** 2022-03-29

**Authors:** Hamid Arazi, Mani Izadi, Hadis Kabirian

**Affiliations:** Department of Exercise Physiology, Faculty of Sport Sciences, University of Guilan, 10th km of Tehran road- Khalij-e-Fars highway, Rasht, 4199843653 Iran

**Keywords:** Older adults, Inactivity, Perception, Physical activity barriers, Interactive effects

## Abstract

**Background:**

Studies examining associations of socio-eco-demographic characteristics with physical activity (PA) participation of older adults have produced inconsistent results. Perceived PA barriers may be a possible explanation for the mixed findings. Therefore, the purpose of this study was to examine the correlation of socio-eco-demographic (SED) characteristics with PA of older adults and the moderation effects of perceived barriers of PA.

**Methods:**

Three hundred eighty-four older adults (≥ 60 years old) were recruited from public places in six different cities. Questions regarding socio-eco-demographic characteristics, PA, and perceived PA barriers were asked, in-person, by two examiners. Ordinal logistic regression models examined the association of socio-eco-demographic characteristics with subjectively measured PA, and the interactive effects of subscales of perceived PA barriers and socio-eco-demographic variables for PA outcomes.

****Results**:**

Significant main effects for PA outcomes were found for education and living status (*P* < 0.01) and college-educated individuals and those were living in their private houses reported higher PA. Also, 24 significant interactive effects of perceived PA barriers by socio-eco-demographic factors were found (*P* < 0.05). Significant moderation effects by all subscales of perceived PA barriers were observed for education and living status. The effect of age for the PA outcomes was moderated by “lack of time”, “fear of injury”, and “lack of skill”. Only “fear of injury” and “lack of time” moderated the effect of gender and marriage for outcome variable, respectively. The effect of employment was moderated by “lack of willpower”, “fear of injury”, “lack of skill” and “lack of resources”.

****Conclusions**:**

Novel evidence revealed that there are moderations by perceived PA barriers for the effect of almost all socio-eco-demographic characteristics. These findings highlight a need to consider older adults’ perspectives and perceptions, when it comes to establish policies for PA participation.

## Introduction

Many factors influence the aging process including genetics, lifestyle factors, and chronic diseases. These factors interact with one another, significantly influencing the way we age. In this context, physical activity/exercise is of the most important lifestyle factors and participation in regular physical activity (PA) is associated with better physiological functions and healthy aging [1], and increasing the level of regular PA is a significant health priority among many countries [2]. Despite well-known benefits of PA and exercise, PA participation rates among older adults population tend to be lower than those observed in younger ages [3, 4].

Of the primary steps in identifying at-risk populations and designing relevant policies and effective preventive programs, especially among older adults, is to determine correlates of participation in PA [5] . Among the most widely studied correlates of overall PA, socio-eco-demographic (SED) variables such as age, gender, employment status, marital status, asset ownership, educational level and income levels were of interest to many authors [6–8], and these variables can help identify population sub-groups at risk for inactivity [9].

In general, it has been shown that advanced age, female gender and being married are negatively correlated with participation in PA among older adults [5, 7, 10, 11]. However, A study found no significant gender differences in PA level among older adults and they speculated that low PA among participants was due to aging [12]. Besides, some studies evidenced the benefits of marriage for increased PA and health-related behaviors [13, 14]. Regarding employment, Smith et al. (2015) showed no significant difference in PA of employed and retired older adults [11], which contradicts the findings of a systematic review demonstrating that physical activity increases after the transition to retirement [15]. The latest can also oppose the adverse effect of advancing age on PA participation, as older adults seem to show greater participation in later ages when they retire.

These mixed findings highlight the need to better understand factors affecting PA behavior among sub-groups of older adult population. Previous studies used socio-ecological models as the theoretical framework to explore the mechanisms that account for differences in PA level by socio-economic status [16–18]. Socio-ecological models posit that behaviour is influenced by multiple levels of factors including intrapersonal (biological, psychological), interpersonal (social, cultural), organisational, community, physio-environmental and policy [19].

Although some of these factors might be, per se, affected by SED, they can also influence PA participation independent of SED. In other words, in socio-ecological models some factors including social, psychological, and cultural factors may play a mediator role in the effect of SED on PA level. However, multiple levels of factors in this model can also be commonplace among older adults regardless of their SED and play a moderator role or interact with the effect of SED on PA.

For instance, Chao et al. [20] investigated exercise adherence among older adults and demonstrated that they deemed the adaptation of moderate PA as time-consuming, as they need to commit certain amount of time both for transportation and for performing exercise. This time commitment might be a greater challenge for those older adults relying on public transportation [21]. In this case, SED may or may not affect (as high socio-economic status does not necessary mean they are or are not using public transportation) older adults’ perception toward time commitment, and thereby influencing PA initiation and adherence.

Authors contended that older adults mostly considered PA as a recreational activity, instead of an effective health intervention [20] and this can be resulted from unclear recommendations given by health practitioners which might be per se compounded by complications among older adults including poor concentration, memory deficits, and dementia [21]. Besides, authors maintained older adults mostly perceived exercise-associated symptoms such as shortness of breath, sweating, and muscle soreness negatively, and as something that harm body instead of benefiting it [20]. Here, older adults’ perception can act somehow independent of their SED.

Therefore, it is speculated that older adults’ perception toward PA participation is associated with their PA level. In this context, some studies on correlates of PA proved that barriers to PA are negatively associated with participation in PA [7, 22]. Besides, in a Delphi study, which allows for gathering the views of experts on an area, determinants of PA were rated by 118 experts and perceived barriers to PA were identified among ten most important factors (out of 73 items) in predicting the initiation of PA [23]. Hence, the inconsistencies in the results of studies on SED correlates of PA may in part be due to potential moderation by older adults’ perceptions toward PA behavior including perceived barriers of PA. However, to our knowledge, no study has been conducted to evaluate the possible moderation effect by barriers of PA on these associations. Therefore, the primary aim of the present study was to examine the correlation of SED characteristics with PA of older adults. Further, as a secondary aim, the moderation effects of perceived barriers of PA on the association of SED variables and PA were sought.

## Methods

### Participants

The procedures were approved by the Institutional Review Committee of the University of Guilan according to the current national laws and regulations and to the latest version of the Helsinki Declaration. The present cross-sectional study recruited participants aged 60 years and over in 2018. Of the 335,313 older adults (based on the latest reports in 2016) 384 residents living in six cities in Guilan province, a northern province in Iran, were recruited using convenience sampling with a 95% confidence interval, margin of error of 5%, and significance level of *p* < 0.05. For each city, several public places including central markets and city centers were chosen where older adults were invited to participate in the study with the following inclusion criteria: permanent resident of the province; lack of any disability significantly restricting PA; and enough oral abilities to respond to questions while questions being asked by examiners. All the participants provided written informed consent to take part in the present study.

### Measurements

The survey activities were in-person and occurred between August and November 2018. Participants were asked by two almost equally experienced examiners to orally respond to questionnaires and responses were recorded by the examiners. The first part of the questionnaire was self-developed and consisted of questions on SED. The second and third parts were questionnaires on PA and perceived barriers of PA, respectively. Further explanation of questions was provided by the examiners when participants needed.

### Socio-eco-demographic characteristics

SED characteristics included age, gender, marital status, education, employment, living status, and income. Due to low response fraction by the participants, income was excluded from SED characteristics. The following binary responses were considered for the characteristics: age, early old age (60–75 years) and late old age (> 75 years); gender, man and woman; marital status, married and single (/divorced/widowed); education, less than college and college and higher; employment, employed and unemployed (/retired); living status, living in private house (where they are landlord or tenant) and living with others (where it is not their own home and they live at their children’s or relatives’ houses, or nursing homes).

### Physical activity

Self-reported PA levels were measured using the first part of the Rapid Assessment of Physical Activity (RAPA) questionnaire. RAPA has been shown to be an easy-to-use, valid measure of physical activity among older adults [24]. Besides, the reliability and validity of RAPA was investigated among Iranian older adults [25]. It was shown to have a good construct validity through confirmatory factor analysis. Besides, test-retest reliability was reported as 0.73 for whole scale. RAPA 1 asks seven questions about light, moderate, or vigorous PA specifying frequencies and durations. According to this measure, five categories of PA were defined as: (1) sedentary (rarely or never do any physical activities), (2) under-active (doing some light or moderate physical activities, but not every week), (3) under-active regular – light activities (doing some light PA every week), (4, 5) under-active regular (doing moderate physical activities every week, but less than 30 min a day or 5 days a week - doing vigorous physical activities every week, but less than 20 min a day or 3 days a week), and (6, 7) active (doing 30 min or more a day of moderate physical activities, 5 or more days a week - doing 20 min or more a day of vigorous physical activities, 3 or more days a week).

### Barriers of physical activity

Barriers to Being Active Quiz (BBAQ) was used to evaluate perceived barriers of PA [26]. The BBAQ is easy to administer and a publicly available measure that targets a large segment of the population. Twenty-one responses are aggregated into seven subscale scores that identify five internal barriers (lack of time, lack of energy, lack of willpower, fear of injury, and lack of skill) and two external barriers (social influence and lack of resources). That is, individual barrier subscales are weighted thorough three questions (a sum of 21 questions) that are rated on a scale from 0 to 3 (very unlikely, somewhat unlikely, somewhat likely, and very likely, respectively) and summed to provide a total score (maximum barrier score of 9) for each barrier subscale. Barriers that receive a total score of five or higher are identified “critical” barriers.

### Statistical analyses

SED characteristics of the participants were presented using percentages. The levels of PA were expressed based on SED characteristics of participants using descriptive statistics and differences in PA by SED characteristics were tested. So that, PA level was expressed using frequencies and percentages for each of two categories in SED characteristics and Mann-Whitney U test was used to investigate differences in PA by SED. For the main analyses, ordinal logistic regression models using Polytomous Universal Model (PLUM), which uses proportional odds model, were conducted. The proportional odds model is commonly used for the analysis of ordinal categorical data and is an extension of classical generalized linear models. It is a generalization of a binary logistic regression model when there are more than two ordinal categories for the response variable [27]. The main effects for the SED characteristics were tested in an initial model, and interactions (interactive effect of SED and PA barriers on PA level) were tested in a subsequent model (models with interactions), with a total of seven models based on seven subscales of BBAQ. All the statistical analyses were performed using SPSS statistical software version 26 (SPSS, Inc., Chicago, IL, USA). Statistical significance was set at *p* ≤ 0.05 (two-tailed).

## Results

Participants’ SED characteristics are presented in Table 1. Table 2 presents differences in PA by SED factors. Significantly higher PA levels were found for participants who were in early old ages (*P* < 0.05), college-educated (and higher) (*P* < 0.01), and those were living in their private houses (*P* < 0.01) compared to their peers. Besides, albeit not significantly (*P* > 0.05), those who were male, being married, and those were employed generally demonstrated greater percentages for higher PA levels (especially in the level 5 of RAPA) other than their counterparts.

**Table 1:** Socio-eco-demographic characteristics of participants

	% of total
Early old age	79.7
Man	51.1
Married	80.2
With college degree	15.3
Employed	15.3
Private house	92.4

**Table 2:** Physical activity by socio-eco-demographic characteristics

	RAPA					Z	Sig
	1	2	3	4	5		
Total	21.4	13	44.8	9.1	11.7		
Age						−2.47	0.013*
Early old age	56 (18.3)	41 (13.4)	141 (46.1)	30 (9.8)	38 (12.4)		
Late old age	26 (33.3)	9 (11.5)	31 (39.7)	5 (6.4)	7 (9)		
Gender						−1.49	0.13
Male	34 (17.3)	27 (13.7)	94 (47.7)	15 (7.6)	27 (13.7)		
Female	48 (25.7)	23 (12.3)	78 (41.7)	20 (10.7)	18 (9.6)		
Marriage						−1.72	0.08
Married	56 (18.2)	42 (13.6)	148 (48.1)	23 (7.5)	39 (12.7)		
Single/Divorced/widow	26 (34.2)	8 (10.5)	24 (31.6)	12 (15.8)	6 (7.9)		
Education						4.55	< 0.001*
< College	75 (23.4)	46 (14.4)	146 (45.6)	26 (8.1)	27 (8.4)		
≥ College	7 (10.9)	4 (6.3)	26 (40.6)	9 (14.1)	18 (28.1)		
Employment						−1.6	0.1
Employed	12 (21.1)	1 (1.8)	30 (52.6)	2 (3.5)	12 (21.1)		
Retired/Unemployed	70 (21.4)	49 (15)	142 (43.4)	33 (10.1)	33 (10.1)		
Living status						−3.2	< 0.001*
Private house	69 (19.4)	46 (13)	162 (45.6)	34 (9.6)	44 (12.4)		
With others	12 (42.9)	4 (14.3)	10 (35.7)	1 (3.6)	1 (3.6)		

RAPA’s subscales are as follows: 1, sedentary; 2, under-active; 3, under-active regular – light activities; 4, under-active regular; 5, active. Values for RAPA are frequencies (percentages) based on 5 levels of physical activity from sedentary to active. “Z” and “Sig” are value of Mann-Whitney U Test and level of Significance. * Significant difference between subclasses of a socio-eco-demographic characteristic in physical activity level

Table 3 presents the associations between SED characteristics and PA, as well as the moderation of these associations by perceived barriers of PA. In the initial model, the main effects for associations between the SED variables and PA outcomes were for education and living status (*P* < 0.01) and those with college (and higher) education and participants living in their private houses reported higher PA. Also, 24 significant perceived barriers of PA by SED factors interactions were found.

**Table 3:** Relation of socio-eco-demographic characteristics and perceived physical activity barriers to physical activity

	Physical Activity			
	OR	LB	UB	Sig
Initial models				
Age	0.403	−0.065	0.871	0.091
Gender	0.209	−0.189	0.608	0.303
Marriage	−0.102	− 0.61	0.406	0.694
Employment	0.391	−0.156	0.937	0.161
Education	−1.188	− 1.706	− 0.669	0.001*
Living status	0.998	0.247	1.749	0.009*
Models with interactions				
Lack of time				
Lack of time is not barrier × Age	1.861	−0.262	3.983	0.086
Lack of time is barrier × Age	0.737	0.182	1.292	0.009*
Lack of time is not barrier × Gender	0.139	−0.643	0.922	0.728
Lack of time is barrier × Gender	0.168	−0.311	0.647	0.493
Lack of time is not barrier × Marriage	−1.273	−2.38	− 0.167	0.024*
Lack of time is barrier × Marriage	0.325	−0.259	0.909	0.275
Lack of time is not barrier × Employment	0.402	−0.476	1.28	0.369
Lack of time is barrier × Employment	0.535	−0.206	1.275	0.157
Lack of time is not barrier × Education	−1.267	−2.3	−0.235	0.016*
Lack of time is barrier × Education	−1.168	−1.796	−0.54	0.001*
Lack of time is not barrier × Living status	1.27	−0.17	2.71	0.084
Lack of time is barrier × Living status	1.215	0.295	2.135	0.01*
Social influence				
Social influence is not barrier × Age	1.78	−0.363	3.923	0.104
Social influence is barrier × Age	0.503	−0.404	1.41	0.277
Social influence is not barrier × Gender	0.042	−0.457	0.54	0.869
Social influence is barrier × Gender	0.308	−0.401	1.017	0.394
Social influence is not barrier × Marriage	−0.118	− 0.835	0.598	0.746
Social influence is barrier × Marriage	−0.244	−1.004	0.516	0.529
Social influence is not barrier × Employment	0.613	−0.048	1.274	0.069
Social influence is barrier × Employment	0.072	−0.951	1.095	0.89
Social influence is not barrier × Education	−1.036	−1.614	−0.459	< 0.001*
Social influence is barrier × Education	0.518	−0.827	1.863	0.45
Social influence is not barrier × Living status	1.51	0.515	2.504	0.003*
Social influence is barrier × Living status	0.403	−0.791	1.598	0.508
Lack of energy				
Lack of energy is not barrier × Age	0.128	− 1.881	2.137	0.9
Lack of energy is barrier × Age	0.399	−0.303	1.102	0.265
Lack of energy is not barrier × Gender	0.234	−0.303	0.771	0.393
Lack of energy is barrier × Gender	0.176	−0.456	0.807	0.586
Lack of energy is not barrier × Marriage	−0.141	− 0.843	0.56	0.693
Lack of energy is barrier × Marriage	−0.154	− 0.926	0.617	0.695
Lack of energy is not barrier × Employment	0.536	−0.216	1.288	0.162
Lack of energy is barrier × Employment	0.282	−0.549	1.112	0.506
Lack of energy is not barrier × Education	−1.478	−2.136	−0.82	< 0.001*
Lack of energy is barrier × Education	−0.292	−1.172	0.588	0.516
Lack of energy is not barrier × Living status	2.33	1.105	3.554	< 0.001*
Lack of energy is barrier × Living status	−0.006	−1.004	0.991	0.99
Lack of willpower				
Lack of willpower is not barrier × Age	−0.448	−2.398	1.502	0.653
Lack of willpower is barrier × Age	0.474	−0.192	1.14	0.163
Lack of willpower is not barrier × Gender	−0.117	− 0.683	0.45	0.687
Lack of willpower is barrier × Gender	0.287	−0.316	0.891	0.35
Lack of willpower is not barrier × Marriage	0.177	−0.638	0.992	0.67
Lack of willpower is barrier × Marriage	−0.414	−1.091	0.263	0.23
Lack of willpower is not barrier × Employment	1.231	0.418	2.044	0.003*
Lack of willpower is barrier × Employment	0.036	−0.739	0.812	0.927
Lack of willpower is not barrier × Education	−1.153	−1.808	−0.497	0.001*
Lack of willpower is barrier × Education	−0.338	−1.242	0.567	0.464
Lack of willpower is not barrier × Living status	2.36	1.215	3.505	< 0.001*
Lack of willpower is barrier × Living status	0.062	−0.998	1.121	0.909
Fear of injury				
Fear of injury is not barrier × Age	−0.677	−2.703	1.349	0.513
Fear of injury is barrier × Age	0.802	0.136	1.467	0.018*
Fear of injury is not barrier × Gender	−0.219	− 0.769	0.332	0.436
Fear of injury is barrier × Gender	0.652	0.019	1.285	0.043*
Fear of injury is not barrier × Marriage	−0.01	−0.757	0.737	0.979
Fear of injury is barrier × Marriage	−0.3	−1.029	0.43	0.421
Fear of injury is not barrier × Employment	0.686	0.013	1.359	0.046*
Fear of injury is barrier × Employment	−0.51	−1.539	0.519	0.331
Fear of injury is not barrier × Education	−1.199	−1.835	−0.563	< 0.001*
Fear of injury is barrier × Education	−0.788	−1.763	0.186	0.113
Fear of injury is not barrier × Living status	2.699	1.422	3.975	< 0.001*
Fear of injury is barrier × Living status	−0.009	−0.998	0.98	0.986
Lack of skill				
Lack of skill is not barrier × Age	−0.067	−2.151	2.017	0.95
Lack of skill is barrier × Age	0.823	0.11	1.537	0.024*
Lack of skill is not barrier × Gender	−0.123	−0.66	0.414	0.654
Lack of skill is barrier × Gender	0.227	−0.419	0.873	0.491
Lack of skill is not barrier × Marriage	−0.053	−0.824	0.718	0.892
Lack of skill is barrier × Marriage	−0.317	−1.019	0.385	0.376
Lack of skill is not barrier × Employment	0.827	0.141	1.512	0.018*
Lack of skill is barrier × Employment	−0.192	−1.135	0.751	0.69
Lack of skill is not barrier × Education	−0.879	−1.481	−0.277	0.004*
Lack of skill is barrier × Education	−0.97	−2.207	0.267	0.124
Lack of skill is not barrier × Living status	1.859	0.719	3	0.001*
Lack of skill is barrier × Living status	0.298	−0.712	1.307	0.563
Lack of resources				
Lack of resources is not barrier × Age	−0.784	−3.565	1.996	0.58
Lack of resources is barrier × Age	0.394	−0.983	1.771	0.575
Lack of resources is not barrier × Gender	0.104	−0.333	0.541	0.64
Lack of resources is barrier × Gender	0.801	−0.363	1.966	0.178
Lack of resources is not barrier × Marriage	−0.074	−0.643	0.495	0.798
Lack of resources is barrier × Marriage	−0.601	−1.852	0.65	0.346
Lack of resources is not barrier × Employment	0.27	−0.313	0.852	0.364
Lack of resources is barrier × Employment	1.897	0.009	3.784	0.049*
Lack of resources is not barrier × Education	−1.067	−1.625	−0.51	< 0.001*
Lack of resources is barrier × Education	−1.606	−3.23	0.019	0.053
Lack of resources is not barrier × Living status	1.238	0.424	2.052	0.003*
Lack of resources is barrier × Living status	−0.237	−2.359	1.884	0.826

Reference categories are as follows: late old age for age; female for gender; single (or divorced or widow) for marriage; retired or unemployed for employment; college education and higher for education attainment; and living with others for living status. OR, LB, and UB refer to odds ratios, lower bound, and upper bound (lower and upper bounds of 95% confidence interval), respectively. * significant effect/interactive effect for physical activity outcome

There were three moderators of age for the PA outcomes (*P* < 0.05). More specifically, when “lack of time”, “fear of injury”, and “lack of skill” were perceived as barriers of PA, there were trends for significance and early older adults showed higher PA levels compared to their peers who were in late old ages. However, no other moderation effect by other subscales of PA barriers was found for age (*P* > 0.05).

On the other hand, only “fear of injury” moderated the effect of gender for outcome variable (*P* < 0.05) and if “fear of injury” was a critical barrier, males would demonstrate upper levels of PA. While, there was only one moderation effect for marriage and it was found for “lack of time” (*P* < 0.05) and among those perceived that “lack of time” is not a PA barrier for them, higher PA levels were shown by singles.

Four moderators of the effect of employment for PA outcomes were found (*P* < 0.05). When “lack of willpower”, “fear of injury”, and “lack of skill” were not perceived as barriers of PA, employed older adults reported greater PA levels. Besides, among those reported “lack of resources” as a barrier, unemployed/retired participants demonstrated lesser PA compared to their employed counterparts.

Interestingly, all subscales of BBAQ significantly moderated (*P* < 0.05) the effect of education for PA outcomes which were regularly in line with the main effect of the education. More specifically, when each of the seven subscales was not perceived as a barrier, college-educated Older Adults demonstrated higher levels of PA compared to their less educated peers. Plus, even among those reported that “lack of time” is a barrier of PA for them, participants without college education reported less PA.

Like education, significant moderation effects of all subscales of BBAQ were found for living status (*P* < 0.05). Among participants reporting “lack of time” as a PA barrier, and those did not perceive “lack of energy”, “lack of willpower”, “fear of injury”, “lack of skill”, “social influence” and “lack of resources” as barriers, older adults were living with others reported lower PA compared to their peers living in their private houses.

## Discussion

The hypothesis of the present study was that evaluating the moderation effect of perceived PA barriers on association between SED characteristics and PA of older adults would help expand upon our knowledge on these associations and explain some of the inconsistencies in the relevant literature. Two out of the six direct associations evaluated between SED factors and PA were significant (Table 3). Besides, approximately 28.5% of the tested interactions between SED factors and perceived PA barriers in relation to PA were found to be significant (Table 3). These findings demonstrate that there is moderation by perceived PA barriers, suggesting there would be further complexities than could be explained only through SED factors.

The results of the present research show that about one fifth of older adults was characterized as sedentary (Table 2). Besides, 44.8% of them found to be under-active regular, meaning that less than the half of them regularly do some light PA every week. Only 11.7% of them met the minimum recommended weekly PA, doing ≥30 min moderate physical activities, 5 or more days a week or doing ≥20 min of vigorous physical activities, 3 or more days a week. Therefore, 88.3% of older adults did not meet the PA level recommended by world leading institutions in health and fitness, including American College of Sports Medicine [28], which is consistent with other findings on PA of Iranian older adults [29]. Further, according to the data available from a review study on PA of older adults, the prevalence of low PA among Iranian older adults found in this study was higher than those observed in other 63 low-to-middle income countries studied previously [30].

In the relevant literature, there have been controversial results on whether increasing age among older adults is associated with PA participation. Resnick et al. (2000) observed no significant differences in the ages of those who exercise regularly and those who did not [31]. Conversely, Janke et al. (2006) studied the leisure habits of older adults and found a significant decrease in PA between the ages of 70 and 80 [32]. In the present study, it was observed that PA participation declines with age. Participants aged 60–75 years demonstrated significantly higher PA levels compared to their older counterparts. Also, in the regression analysis, there was a tendency (albeit not significantly) towards significant effect of age for PA outcomes.

The results showed that there are significant differences in PA participation of older adults when they were compared in relation to education attainment and living status. More specifically, those with college education (and higher) and those living in their private houses reported significantly higher PA levels. Similarly, significant main effects for PA outcomes were found by education and living status in the regression analysis. Previous findings on older adults have consistently explained that participation in PA is lower among those who are less educated [32–35]. Besides, the type of residency and local culture might be associated with PA behavior. Walking has been reported to be less among those living in a rented accommodation than that of home owners [36]. However, it was shown that the risk of inactive lifestyle was 1.47 times higher for women living alone [37]. Therefore, further research seems to be needed to better explain the relationship between type of residency and its culture with PA.

It is well established that of the chief PA barriers across old ages are health problems and associated fear of injury/falling. Mathews et al. (2010) investigated the perceived PA in a multicultural older adults population and found health problems, fear of falling, and inconvenience as common barriers [38]. Additionally, our findings demonstrated that when “fear of injury” is perceived as a critical barrier, younger older adults would participate in PA more than their older peers. In agreement with these results, a review study highlighted that when studying PA of oldest old it is necessary to focus on a number of factors among which fear of injury receives an especial attention [39]. Also, “lack of skill” moderated the effect of age for PA and when it is perceived as a barrier, older adults would demonstrate lower PA participation. This evidence together with previously mentioned findings in comparison of age classifications necessitate the implementation of PA policies and priorities specifically established for older adults, considering their fears, needs, and preferences.

Although no significant effect of gender for PA outcome was found, there was a moderation by “fear of injury” and when it is thought to be a potential barrier, women would participate in PA less than their male counterparts. This finding is consistent with the results of Gobbi et al. (2012) among Brazilian older adults, reporting the “fear of falling and being hurt” as the most important barrier among women [40]. Similarly, through a research on Icelandic population, low self-efficacy and fear of falling have been shown to be significant limiting factors for many older women and suppressed their PA participation [41]. Gobbi et al. (2012) also explained that women reported significantly more barriers than men in all 60–69, 70–79, and ≥ 80 year age groups, even though it was marginally significant among the last two categories [40]. Therefore, the evidence on older adults from different geographical locations and cultures shows that women commonly face further perceived limiting factors when compared to men and fear of injury/falling is amongst the most significant barriers.

Previous research supports the benefits of marriage on health and the correlation between being married and health-related behaviors, and shows that spousal concordance in health behaviors extends into later life [14]. However, the evidence on PA remains to be inconsistent. The majority of studies indicated that marriage is inversely correlated with PA [7]. On the other hand, one study opposes these findings by showing that getting married is positively associated with PA [42]. So, this lack of agreement is likely to be moderated by other factors than age and gender. In the present research, “lack of time” was the single moderator for PA outcomes and among those perceived that “lack of time” is not a PA barrier for them, higher PA levels were shown by singles. Therefore, we are still unable to draw conclusions on this moderation and further research needs to be elucidated.

The effect of employment for the PA outcomes was moderated by four subscales of BBAQ. Among those reported that “lack of willpower”, “fear of injury”, and “lack of skill” are not PA barriers, employed older adults reported greater PA participation. Similar results were seen when “lack of resources” was a barrier. The results of the present study on the moderation of perceived PA barriers on the effect of employment for PA outcomes appears to be controversial and we are not able to draw any solid conclusion on. One possible explanation, especially for “lack of resources”, would be the few numbers of employed participants at the age of 60 and over, as seen in the present study (by only ~ 15%). In this regard, Sawchuk et al. (2011) examined the reliability of BBAQ among older adults. They reported Cronbach’s alpha as 0.87 for total scale. Nevertheless, it was shown to be 0.45 for “lack of resources” [43]. So, this would have affected the results. Altogether, there seem to be moderation effects by perceived PA barriers on PA.

Regarding education attainment and living status, there was moderation by each of the BBAQ subscales, generally being consistent with the main effects of the education and living status. The effect of education for PA involvement would be explained through several assumptions. First, more educated participants would have further knowledge on the advantages of PA. Second, this people may represent better socioeconomic status, and then, better access to resources for being physically active. Lastly, more educated people may be exposed to more PA programs through educational system [3]. That could be the case for those living in their own private houses and these individuals may represent a higher socioeconomic stratum. Besides, they would generally possess a better physical health compared to those living in other places including nursing homes, which in turn, could significantly affect older adults’ participation in PA programs.

The present research had several strengths and limitations. First, this is the first study to investigate the moderation of perceived barriers of PA on the correlation between SED characteristics and PA in older population. Besides, we took barriers from various domains using a validated questionnaire on PA of older adults. Therefore, this multifaceted approach helps us better understand the perceived PA barriers among older adults and the moderation they might have on the effect of SED characteristics for PA participation. As the limitations, due to funding restrictions, it was not possible to include larger population and we collected data only from the minimum sample size suggested by previous studies. While, a bigger sample size commonly results in more confident findings. Another limitation was the lack of calculation of the inter-reliability of the examiners.

Additionally, as a result of low response fraction, we were unable to report income which is of the most important factors in studies on SED characteristics and this could have unraveled further on the effects and relationships investigated in this study. Lastly, we did not account for the possible consequences of running multiple comparisons. To avoid rejection of null hypothesis too readily, adjustments for multiple comparisons are recommended. These adjustments, such as alpha error corrections, reduce Type I error for null associations but they elevate Type II error, as an overconservative approach is considered and in these cases true relationships might be rejected. As we cannot argue that the first explanation for the significant relationships found in the present study is “chance” and we followed a theoretical framework for our hypotheses, correction of alpha is not desirable [44]. However, due to possibility of Type I error inflation in multiple comparisons, especially regarding moderation effects, further studies need to be conducted to draw solid conclusions.

## Conclusion

In conclusion, our results confirmed the positive association of education and PA involvement showed by a great majority of previous studies. Even though our findings demonstrated a positive correlation between living in private house and PA participation, further studies seem to be needed to reach a consensus. Also, novel evidence showed that there are moderations by perceived PA barriers for the effect of almost all SED characteristics on PA participation among which the “fear of injury” for older and female participants, and the “lack of resources” for unemployed/retired individuals were more evident and meaningful.

Our findings suggest that older adults’ perspectives and perception remain central to discussions on the design of PA programs. Therefore, before making decisions and policies on the PA of older population, it is hugely needed to consider potential obstacles for participation in PA programs perceived by older adults, together with raising awareness regarding health benefits of taking part in recommended physical activities and minimizing the risk of physical inactivity, and how to minimize the risks of injury during PA.

## Data Availability

The dataset of the study is available from the corresponding author upon reasonable request.

## References

[1] Mazzeo R, Cavanagh P, Evans W, Fiatarone M, Hagberg J, McAuley E (1998). Exercise and physical activity for older adults: American College of Sports Medicine position stand. Med Sci Sports Exerc.

[2] Commonwealth Department of Health and Family Services. Developing an Active Australia: A framework for action for physical activity and health. Canberra: Commonwealth Government of Australia; 1998.

[3] Haley C, Andel R (2010). Correlates of physical activity participation in community-dwelling older adults. J Aging Phys Act.

[4] Lohne-Seiler H, Hansen BH, Kolle E, Anderssen SA (2014). Accelerometer-determined physical activity and self-reported health in a population of older adults (65–85 years): a cross-sectional study. BMC Public Health.

[5] Biernat E, Tomaszewski P (2011). Socio-demographic and leisure activity determinants of physical activity of working Warsaw residents aged 60 to 69 years. J Hum Kinet.

[6] Bauman AE, Reis RS, Sallis JF, Wells JC, Loos RJ, Martin BW, Lancet Physical Activity Series Working Group (2012). Correlates of physical activity: why are some people physically active and others not?. Lancet.

[7] Trost SG, Owen N, Bauman AE, Sallis JF, Brown W (2002). Correlates of adults’ participation in physical activity: review and update. Med Sci Sports Exerc.

[8] Stamatakis E, Grunseit AC, Coombs N, Ding D, Chau JY, Phongsavan P, Bauman A, SEEF Project (2014). Associations between socio-economic position and sedentary behaviour in a large population sample of Australian middle and older-aged adults: the social, economic, and environmental factor (SEEF) study. Prev Med.

[9] Mitáš J, Cerin E, Reis RS, Conway TL, Cain KL, Adams MA, Schofield G, Sarmiento OL, Christiansen LB, Davey R, Salvo D, Orzanco-Garralda R, Macfarlane D, Hino AAF, de Bourdeaudhuij I, Owen N, van Dyck D, Sallis JF (2019). Do associations of sex, age and education with transport and leisure-time physical activity differ across 17 cities in 12 countries?. Int J Behav Nutr Phys Act.

[10] van Stralen MM, De Vries H, Mudde AN, Bolman C, Lechner L (2009). Determinants of initiation and maintenance of physical activity among older adults: a literature review. Health Psychol Rev.

[11] Smith L, Gardner B, Fisher A, Hamer M (2015). Patterns and correlates of physical activity behaviour over 10 years in older adults: prospective analyses from the English longitudinal study of ageing. BMJ Open.

[12] Milanović Z, Pantelić S, Trajković N, Sporiš G, Kostić R, James N (2013). Age-related decrease in physical activity and functional fitness among elderly men and women. Clin Interv Aging.

[13] King AC, Kiernan M, Ahn DK, Wilcox S (1998). The effects of marital transitions on changes in physical activity: results from a 10-year community study. Ann Behav Med.

[14] Li K-K, Cardinal BJ, Acock AC (2013). Concordance of physical activity trajectories among middle-aged and older married couples: impact of diseases and functional difficulties. J Gerontol B Psychol Sci Soc Sci.

[15] Barnett I, van Sluijs EM, Ogilvie D (2012). Physical activity and transitioning to retirement: a systematic review. Am J Prev Med.

[16] Ball K, Timperio A, Salmon J, Giles-Corti B, Roberts R, Crawford D (2007). Personal, social and environmental determinants of educational inequalities in walking: a multilevel study. J Epidemiol Community Health.

[17] Cerin E, Leslie E (2008). How socio-economic status contributes to participation in leisure-time physical activity. Soc Sci Med.

[18] Kamphuis C, Van Lenthe FJ, Giskes K, Huisman M, Brug J, Mackenbach JP (2009). Socioeconomic differences in lack of recreational walking among older adults: the role of neighbourhood and individual factors. Int J Behav Nutr Phys Act.

[19] Champion VL. Skinner CS. The health belief model. In Glanz K, Rimer BK, Viswanath K. (eds), Health Behavior and Health Education: Theory, Research, and Practice. San Francisco: Jossey-Bass; 2008. p. 45–65.

[20] Chao D, Foy CG, Farmer D (2000). Exercise adherence among older adults: challenges and strategies. Control Clin Trials.

[21] Schutzer KA, Graves BS (2004). Barriers and motivations to exercise in older adults. Prev Med.

[22] Bauman AE, Sallis JF, Dzewaltowski DA, Owen N (2002). Toward a better understanding of the influences on physical activity: the role of determinants, correlates, causal variables, mediators, moderators, and confounders. Am J Prev Med.

[23] van Stralen MM, Lechner L, Mudde AN, de Vries H, Bolman C (2010). Determinants of awareness, initiation and maintenance of physical activity among the over-fifties: a Delphi study. Health Educ Res.

[24] Topolski TD, LoGerfo J, Patrick DL, Williams B, Walwick J, Patrick MMB. Peer reviewed: the Rapid Assessment of Physical Activity (RAPA) among older adults. Prev Chronic Dis. 2006;3(4):A118. PMC1779282 16978493

[25] Khajavi D, Khanmohamadi R (2015). Predicting depressive symptoms of the elderly according to physical activity level and demographic characteristics: examining the role of age and gender. J Motor Learn Movement.

[26] Centers for Disease Control and Prevention. Barriers to Being Physically Active Quiz. Available online: https://www.cdc.gov/diabetes/ndep/pdfs/8-road-to-health-barriers-quiz-508.pdf. Accessed Aug 2018.

[27] Liu X (2009). Ordinal regression analysis: fitting the proportional odds model using Stata, SAS and SPSS. J Mod Appl Stat Methods.

[28] Garber CE, Blissmer B, Deschenes MR, Franklin BA, Lamonte MJ, Lee I-M, et al. Quantity and quality of exercise for developing and maintaining cardiorespiratory, musculoskeletal, and neuromotor fitness in apparently healthy adults: Guidance for prescribing exercise. Special communications: Position stand. Med Sci Sports Exerc. 2011;43(7):1334–1359. 10.1249/MSS.0b013e318213fefb. 10.1249/MSS.0b013e318213fefb 21694556

[29] Eshaghi SR, Shahsanai A, Ardakani MM. Assessment of the Physical Activity of Elderly Population of Isfahan, Iran. J Isfahan Med Sch. 2011;29(147):939–46.

[30] Vancampfort D, Koyanagi A, Ward PB, Rosenbaum S, Schuch FB, Mugisha J, Richards J, Firth J, Stubbs B (2017). Chronic physical conditions, multimorbidity and physical activity across 46 low-and middle-income countries. Int J Behav Nutr Phys Act.

[31] Resnick B, Palmer MH, Jenkins LS, Spellbring AM (2000). Path analysis of efficacy expectations and exercise behaviour in older adults. J Adv Nurs.

[32] Janke M, Davey A, Kleiber D (2006). Modeling change in older adults’ leisure activities. Leis Sci.

[33] Droomers M, Schrijvers CTM, Mackenbach J (2001). Educational level and decreases in leisure time physical activity: predictors from the longitudinal GLOBE study. J Epidemiol Community Health.

[34] Mouton CP, Calmbach WL, Dhanda R, Espino DV, Hazuda H (2000). Barriers and benefits to leisure-time physical activity among older Mexican Americans. Arch Fam Med.

[35] Weiss DR, O'Loughlin JL, Platt RW, Paradis G (2007). Five-year predictors of physical activity decline among adults in low-income communities: a prospective study. Int J Behav Nutr Phys Act.

[36] Dawson J, Hillsdon M, Boller I, Foster C (2007). Perceived barriers to walking in the neighbourhood environment and change in physical activity levels over 12 months. Br J Sports Med.

[37] Jeong S, Il Cho S. Effects of living alone versus with others and of housemate type on smoking, drinking, dietary habits, and physical activity among elderly people. Epidemiol Health. 2017;39(34):1–7. 10.4178/epih.e2017034 PMC5675988 29121710

[38] Mathews AE, Laditka SB, Laditka JN, Wilcox S, Corwin SJ, Liu R, Friedman DB, Hunter R, Tseng W, Logsdon RG (2010). Older adults’ perceived physical activity enablers and barriers: a multicultural perspective. J Aging Phys Act.

[39] Baert V, Gorus E, Mets T, Geerts C, Bautmans I (2011). Motivators and barriers for physical activity in the oldest old: a systematic review. Ageing Res Rev.

[40] Gobbi S, Sebastiao E, Papini CB, Nakamura PM, Valdanha Netto A, Gobbi LTB (2012). Physical inactivity and related barriers: a study in a community dwelling of older brazilians. J Aging Res.

[41] Bjornsdottir G, Arnadottir SA, Halldorsdottir S (2012). Facilitators of and barriers to physical activity in retirement communities: experiences of older women in urban areas. Phys Ther.

[42] King AC, Castro C, Wilcox S, Eyler AA, Sallis JF, Brownson RC (2000). Personal and environmental factors associated with physical inactivity among different racial–ethnic groups of US middle-aged and older-aged women. Health Psychol.

[43] Sawchuk CN, Russo JE, Bogart A, Charles S, Goldberg J, Roy-Byrne P, et al. Peer reviewed: barriers and facilitators to walking and physical activity among American Indian elders. Prevent Chronic Dis. 2011;8(3):A63. PMC3103568 21477503

[44] Rothman KJ (1990). No adjustments are needed for multiple comparisons. Epidemiology.

